# VIAE-Net: An End-to-End Altitude Estimation through Monocular Vision and Inertial Feature Fusion Neural Networks for UAV Autonomous Landing

**DOI:** 10.3390/s21186302

**Published:** 2021-09-20

**Authors:** Xupei Zhang, Zhanzhuang He, Zhong Ma, Peng Jun, Kun Yang

**Affiliations:** 1 Xi’an Microelectronics Technology Institute, Xi’an 710065, China; zxp771tuantuan@163.com (X.Z.); hzz771@163.com (Z.H.); 2 Sichuan Tengden Technology Co., Ltd., Chengdu 610037, China; pnjun@163.com (P.J.); greyshadowii@hotmail.com (K.Y.); 3 School of Information and Communication Engineering, University of Electronic Science and Technology of China, Chengdu 611731, China

**Keywords:** altitude estimation, visual-inertial data fusion, self attention, UAV autonomous landing

## Abstract

Altitude estimation is one of the fundamental tasks of unmanned aerial vehicle (UAV) automatic navigation, where it aims to accurately and robustly estimate the relative altitude between the UAV and specific areas. However, most methods rely on auxiliary signal reception or expensive equipment, which are not always available, or applicable owing to signal interference, cost or power-consuming limitations in real application scenarios. In addition, fixed-wing UAVs have more complex kinematic models than vertical take-off and landing UAVs. Therefore, an altitude estimation method which can be robustly applied in a GPS denied environment for fixed-wing UAVs must be considered. In this paper, we present a method for high-precision altitude estimation that combines the vision information from a monocular camera and poses information from the inertial measurement unit (IMU) through a novel end-to-end deep neural network architecture. Our method has numerous advantages over existing approaches. First, we utilize the visual-inertial information and physics-based reasoning to build an ideal altitude model that provides general applicability and data efficiency for neural network learning. A further advantage is that we have designed a novel feature fusion module to simplify the tedious manual calibration and synchronization of the camera and IMU, which are required for the standard visual or visual-inertial methods to obtain the data association for altitude estimation modeling. Finally, the proposed method was evaluated, and validated using real flight data obtained during a fixed-wing UAV landing phase. The results show the average estimation error of our method is less than 3% of the actual altitude, which vastly improves the altitude estimation accuracy compared to other visual and visual-inertial based methods.

## 1. Introduction

The process of estimating the relative altitude between the UAV and a specific area is usually known as altitude estimating. For decades, altitude has been one of the crucial flight parameters of UAV navigation in various applied fields, such as automatic landing and takeoff [1,2], obstacle avoidance, precise localization, and flight cruising. Therefore, improved altitude estimation techniques are intensely explored. Existing altitude estimation methods rely heavily on a Global Positioning System (GPS), Inertial Navigation System (INS), barometric altimeter and other active ranging sensors. However, owing to complex application environments, a GPS signal can be easily interfered with or blocked, thus, altitude estimation by receiving GPS signals may not be possible. On the other hand, as stated in [3,4], all INS methods suffer from integration drift, and it cannot be eliminated unless another measurement from a different sensor has been introduced. Thus, in most cases, INS methods need to compensate with other active ranging sensors to estimate altitude. However, active range sensors, such as radar altimeters, laser rangefinders, and ultrasonic altimeters commonly have a limited measurement range or only have low precision for long-range altitude measurement. Moreover, the barometric altimeter is the conventional altimetric sensor for UAVs in high-altitude environments, but when the UAV is close to the ground there are too many factors (such as weather, local air temperature and humidity) which impact their precision of altitude estimation. Altitude estimation provided by these methods is typically inaccurate [5] or needs expensive and high-power consumption equipment to guarantee the estimation precision. At this time, the advantages of visual-based altitude estimation methods are particularly important.

**Figure 1:**
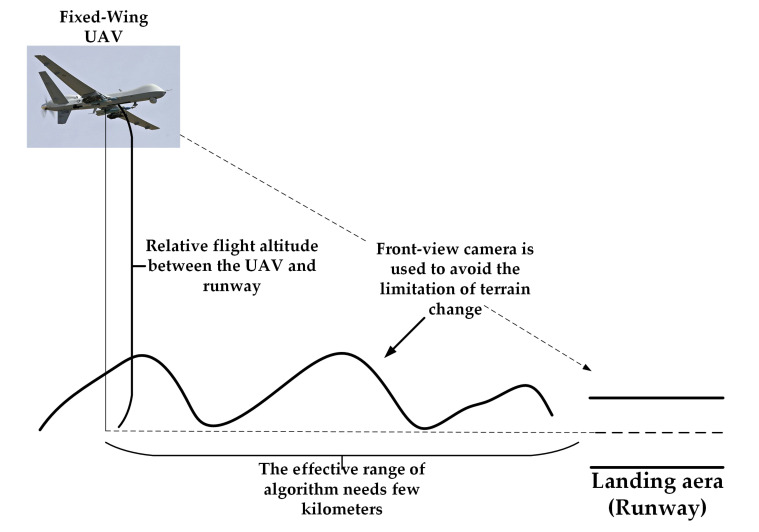
The vision-based altitude estimation problem for fixed-wing UAVs.

In recent years, vision-based altitude estimation solutions have been attractive since they are passive, low-cost, and applicable in a GPS denied environment. As shown in Figure 1, for fixed-wing UAVs, it is not possible to hover in the air to detect the landing area and land slowly as VTOL (vertical take-off and landing) UAVs do [1,2,6]. Therefore, for fixed-wing UAVs, the vision-based altitude estimation algorithm aims to detect the visual features of the landing area using forward-looking vision sensors, and then use kinematic and camera models to estimate the flight altitude of the UAV relative to the landing area. However, from the imaging principle of a monocular camera, we cannot know the actual size of an object when the monocular camera captures only one image. Researchers also call this problem “scale ambiguity”. When the monocular camera is moving, the error caused by scale ambiguity will keep accumulating to lead to a scale drift problem [7,8,9]. This problem makes it difficult to obtain realistic flight altitude estimation results directly. On the other hand, although stereo cameras can solve the scale drift problem to some extent, a limited baseline length and camera resolution will also prevent stereo cameras from achieving accurate measurements under the demand of long-range detection, and in addition, the design of stereo camera systems has problems, such as a high cost and large computational effort [10,11,12]. Especially, an accurate estimation is always required throughout the landing phase of the fixed-wing UAV, from long distance to close range. However, a stereo camera with a long baseline, which is good at long-distance estimation, may be incapable of detecting near objects because the overlapped area of the FOV (field of view) of both cameras is quite small. To solve this ‘blind spot’ problem, a specialized system with a flexible baseline of the stereo camera needs to be designed from the aspect of hardware and software. Therefore, existing algorithms usually integrate monocular vision information with other external information (e.g., IMU measurement information or some known information from the real world) to solve the monocular vision scale drift problem [13,14,15,16,17]. However, fusing the visual and the other relevant information to build altitude estimation models is still a complex and challenging problem. On the one hand, physical model-based reasoning approaches require extremely complex and precise priors (e.g., data synchronization and sensor calibration, etc.) [18,19,20,21], otherwise the accuracy of the built altitude estimation model will be limited. On the other hand, recent approaches that use deep neural networks to learn the complex UAV kinematic models in data-driven ways have shown great potential for UAV state estimation (such as altitude, pose and location, etc.) [22,23,24]. However, a natural concern for this kind of method is the necessity to acquire large amounts of data. This can be very costly and difficult, especially when these data have to be acquired from interactions with the real world, as in the case in UAVs. Therefore, this type of algorithm is rarely applied to real scenarios at present.

Considering the issues above and inspired by previous works [25,26,27,28,29], this paper explores the integration of physical-based reasoning into modern CNN-LSTM-based models and the fusion of different types of features to further improve altitude estimation for fixed-wing UAV landing. Therefore, aiming at the above situation, we propose a novel end-to-end neural network named the visual-inertial altitude estimation network (VIAE-Net). The main contributions are summarized below.

(1) We proposed a novel altitude estimation method that integrated a physical-based model into a deep neural network architecture to build a more robust and accurate altitude estimation model with visual and inertial data sequences. The current physical-based or learning-based methods cannot balance broad applications and performance, as well as data efficiency and a large requirement of training data. However, the physical reasoning we introduced into the network will be a driving stimulant, which can not only broaden the scope of applications and improve data efficiency of the CNN-LSTM-based altitude model, but also achieve a high-precision estimation for an extended range in various real scenarios.
(2) Based on several appropriate assumptions, we designed a physical-based altitude model consisting of an image model of a monocular camera with kinematic principles. The model can not only ideally reveal the functional relations between the altitude values, the known information from sensors and their relations, but also simplify the training process by taking it as a part of the initial model of the neural network.
(3) We present a novel feature fusion module for the visual and inertial information, which uses a self-attention mechanism to map different features into the same feature space. Benefitting from this, the neural network can perceive the complex data association between the visual-inertial data sequences and the altitude model to improve the robustness and precision of the altitude estimation results.

Owing to these contributions, the proposed method obtains an average estimation error that is less than 1% of the actual UAV altitude in real scenes and is superior to existing height estimation methods by 3–10 times. Moreover, the proposed method shows a lower peak error than other compared approaches, which means that the performance of the proposed method is more robust for input data with a motion blur, jitter, or drift problem.

The rest of this work is structured as follows: in the next section, some related visual-based altitude estimation methods are presented. In Section 3, our proposed method VIAE-Net is introduced. Section 4 provides the experimental results and analysis of our method and the other methods on the real scenario dataset. Finally, conclusions are drawn in Section 5.

## 2. Related Work

For decades, visual-based altitude estimation has received a significant amount of attention in computer version and robot navigation. At the early stage, altitude estimation was traditionally considered as an ego-motion modeling task, which uses the camera model, a reference in the real scenario, and kinematic modeling of the UAV to build the functions to calculate the relative altitude between the UAV and the specific area. To solve scale drift [7,8] and motion blur [30,31] from the monocular camera, recent studies show that inertial information from the IMU on board can significantly improve the modeling of the relative altitude. In recent years, neural network methods show that their data-driven modeling capability has a great perspective on altitude or pose estimation. Thus, the mainstream methods for visual altitude estimation can be roughly categorized as model-based methods and deep learning methods. In this section, we briefly review these related works and discuss the inspiration from these methods.

**Physical-based methods:** As mentioned above, pure monocular vision-based methods will face scale drift caused by the imaging principle of the monucular camera. Thus, before these methods estimate altitude from image sequences, a landmark with a physical length needs to be established for altitude modeling. At present, most of the small rotor UAVs or drones usually set a marker on the landing area [14,32,33] for the algorithms to estimate the altitude and position of the UAV. These methods show a high accuracy altitude estimation (<20 cm) at a close range (the UAVs are 20–30 m high above the ground) and need to keep the camera downward to the ground for the entire process. Therefore, these methods cannot be used in a fixed-wing UAV landing scenario, because it requires a larger range of estimation and the kinematic model of fixed-wing UAVs is more complex since their speed is much higher than VTOL UAVs. In addition, fixed-wing UAVs cannot hover in the sky for smooth landing as the VTOL UAV does. On the other hand, although the stereo camera method can solve the scale drift problem in some limited conditions [10], their precision and effectiveness of altitude estimation is also limited by the length of the camera baseline, since the mounting space on the UAV is limited. For a long-range estimation task, it is necessary to design a stereo camera with a variable baseline which is complex and expensive. Moreover, the computational cost of the stereo method is much higher than the physical-based monocular methods. Obviously, these strict conditions will limit the application of these methods in different scenarios.

Except for the above, these methods have common drawbacks: (1) the jitter and fast motion of UAVs will lead to motion blur during image capture processing which will impact on the quality of the images and cause errors in altitude estimation. (2) traditional vision-based UAV altitude estimation usually applies simple linearization of the UAV and camera kinematics to simplify the estimation problem, which is a complex nonlinear problem for the real landing phases. This idealized processing will limit the precision of the altitude estimation.

To deal with the missing visual information caused by UAV high-speed motion and jitter, the strategy of visual and inertial data fusion has received considerable attention in altitude and pose estimation for UAVs [21,34,35,36,37]. In state-of-the-art methods, visual and inertial data fusion is usually achieved through building a filter-based or optimization-based procedure, such as the Constraint Kalman Filter in [38] and the iSAM2 [39] in [40]. The experimental comparison in [35] shows that the state-of-the-art methods achieved centimeter-level estimation results in small-scale indoor scenes. However, these extraordinary precision results are based on manual calibration and synchronization before the fusion of visual and inertial data. On the other hand, the image and IMU measurement data are always captured in different frequencies, which is an essential factor for data fusion [21,37,38]. In other words, calibration and synchronization are crucial for building the altitude estimation model with visual and inertial information. Therefore, without calibration and synchronization, huge errors will be introduced into the altitude model and impact the estimation precision.

**Deep learning methods:** In recent years, deep learning algorithms have demonstrated their powerful capabilities in representing different types of data and fusing nonlinear features. Thus, researchers have shown a lot of interest in applying learning algorithms to the problem of real-time position and pose estimations for UAV automatic control and landing [22,41,42]. In the methods of [43], the authors design a novel framework for monocular visual odometry based on a convolutional neural network (CNN) and recurrent neural network (RNN) to achieve a 6-DOF pose estimation for the camera. They exploit the CNN to obtain the high-level features (such as photometric consistency) from images and use the RNN to explore the temporal features between images. Inspired by this work, the authors in [44] introduced the Bi-directional LSTM (Bi-LSTM), instead of the RNN to increase the capability of exploring the temporal information for long-term image sequences, and the experimental results show that it achieves more accurate estimation results than the method in [43]. The experimental results of both the methods demonstrated that the CNN-LSTM architecture can obtain rich visual features and temporal features from the sequence data. To some extent, these features can help the network learn a more accurate model to represent the altitude or pose of the UAVs. However, these methods drawn from the photometric and temporal consistency in monocular image sequences still cannot solve the scale drift problem. Thus, the estimate accuracy should be further improved, which is far behind the state-of-the-art traditional visual-inertial method [35]. Recently, existing works on applying CNN-LSTM networks to solve visual-inertial based UAV position and pose estimation have shown competitive performances in accuracy and robustness [29,41,45]. In [41], the authors present the first system for visual-inertial aided navigation based on deep learning networks. They used the CNN network to extract and encode visual features from the image sequence and used the LSTM network to encode the IMU measurements sequence. Then, they directly concatenated these encoded features and fed them into a physical-based layer operation for pose regression. The performance of this work shows the potential of deep learning to solve complex kinematic modeling and state estimation problems. However, it is not taken into account that the feature representations of image and IMU data are too different to join directly. Therefore, the experimental results are still much worse than the physical visual-inertial methods with manual calibration and synchronization. To improve the weakness, the authors of [29] introduce two different strategies to deal with the fusion of inertial and visual features. The authors in [45] used geometric constraints to lead the fusion of different features by adding a stereo supervision network into the visual-inertial framework. However, these two methods are designed for automatic driving of ground vehicles and their great performances are deeply dependent on the large amount of their training data. Table 1 summarizes the main properties of the different types of methods.

In summary, physical-based methods, which aim to build an explicit model to estimate the altitude, have wide applicability and impressive data efficiency due to their universal underlying physical rules. However, these methods can hardly converge to a good enough local optimum point under the circumstances of low-quality data preprocessing or invalid kinematic modeling. In contrast, deep learning methods show their superior capability of data perception by employing a large number of parameters to implicitly represent the altitude modeling with a high-quality local optimum after the model has been sufficiently trained. However, it is worth noting that their remarkable performance is on the basis of large amounts of training datasets which are usually hard to obtain, and these implicit models limit the scope of application due to the lack of general physics-based reasoning. In addition, purely monocular vision methods are incapable of achieving precise height estimation under some severe conditions, such as motion blur. In addition, only relative height estimations can be obtained due to the lack of scale information, unless other sensors (such as IMU measurements) or other known information in the real world is introduced. On the other hand, the great performance of the physical-based visual-inertial methods require precise physical modeling and data preprocessing which is difficult to perfectly satisfy in practical applications. In contrast, learning based visual-inertial methods can solve these problems in a data-driven manner. However, such methods require a data fusion mechanism to explore the high-level data associations between different types of data to achieve accurate modeling of the altitude estimation problem.

## 3. Materials and Methods

**Figure 2:**
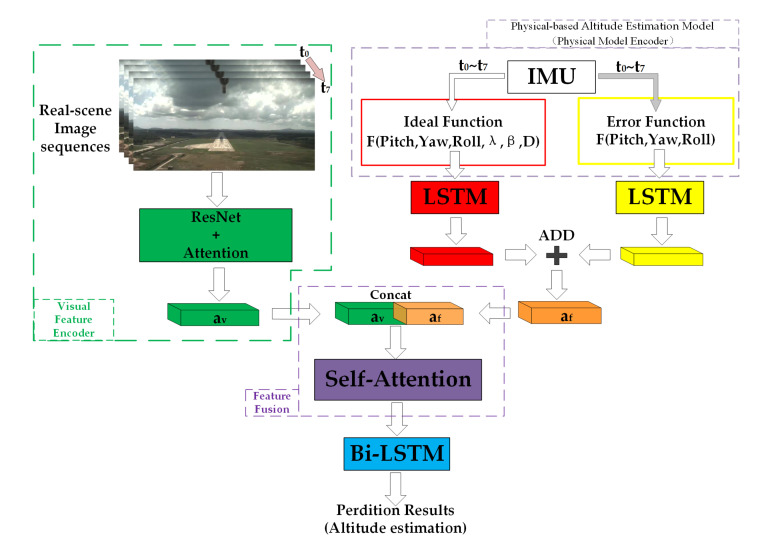
The proposed VIAE-Net architecture for altitude estimation.

In this section, we introduce the end-to-end neural network architecture for altitude estimation, which is the foundation for our proposed framework. Figure 2 shows an overview of the architecture, consisting of a physical-based altitude estimation model, a visual feature encoder, a feature fusion module, and a temporal feature extraction and altitude regression module. The physical-based altitude estimation model is the first difference between our framework and existing methods [41,44]. As we mentioned before, the integration of the physical model can help the neural network to reduce the required amount of training data. It includes an ideal function to represent the altitude estimation model with visual and inertial information, whereas the error function uses the IMU measurements to represent the modeling error which complements the ideal function. The sum of the ideal function and the error function is the complete physical-based altitude estimation model. We use $a_{f}$ to represent the temporally encoded features of the physical-based altitude estimation model using the LSTM network. The visual feature encoder aims to extract the visual feature $a_{v}$ from the region of interest in images using a CNN network. Instead of directly concatenating the visual-inertial features, which ignores the different physical meaning under the different types of data, a novel feature fusion module is designed for the proposed network architecture. Inspired by the previous work [46,47], the feature fusion module uses the self-attention mechanism to obtain the similarity of different types of features, then uses the similarity to reweight the feature map to achieve the feature fusion. In this work, it is designed to fuse the visual features with physical altitude model features to help the network perceive the implicit data (feature) association between these different types of features. This is a critical part of the proposed method. After the feature fusion module, the temporal feature extraction and altitude regression module utilises the Bi-LSTM to extract the temporal information from the fused feature to obtain the altitude estimation results. Our model takes roughly synchronized monocular RGB image sequences and raw IMU measurements as the network input. The proposed approach aims to learn a function F(·) that predicts the corresponding altitude of the input data.

### 3.1. Physical-Based Altitude Estimation Model (Physical Model Encoder)

As we mentioned above, the traditional vision-based methods need to build the altitude estimation model based on the intrinsic camera projection transformation, and camera kinematics. However, the altitude estimation in the real landing phases is a complex nonlinear problem that is difficult to precisely represent by these parameters. The idealized processings, such as simple linearization and approximation, are unavoidable during the modeling process. In contrast, theoretically, any function can be fitted by a two-layer neural network if the number of neurons and datasets are sufficiently large. However, it is difficult to train a network to solve high-dimensional problems with limited data. Therefore, to reduce the requirement of the training data and the complexity of training, the proposed method builds an ideal physical model to represent the altitude estimation first, and then uses the neural network to learn an error function and the linear composite between the ideal function and the error function from the images and corresponding IMU measurements. Thus, the altitude estimation model formulation can be represented as:

(1) $$Altitude=F\left(V_{1},\ldots ,V_{N},I_{1},\ldots ,I_{N}\right)=f\left(V_{1},\ldots ,V_{N},I_{1},\ldots ,I_{N}\right)+\varepsilon \left(I_{1},\ldots ,I_{N}\right)$$

where $V_{1},\ldots ,V_{N}$ represents the visual information from the monocular camera and $I_{1},\ldots ,I_{N}$ represents the inertial information from the IMU on the UAV.

#### 3.1.1. Ideal Function

To simplify the modeling process, we make four assumptions:

(1) The camera and IMU are located in the same location on the UAV, and the coordinate systems of these two sensors are coincident.
(2) The camera position can represent the UAV position.
(3) The runway can be observed completely on the image plane. The actual width is known and the bottom angles λ and β can be obtained by the runway detection method.
(4) The yaw angle is the relative angle between the UAV heading angle and the runway orientation angle.

**Figure 3:**
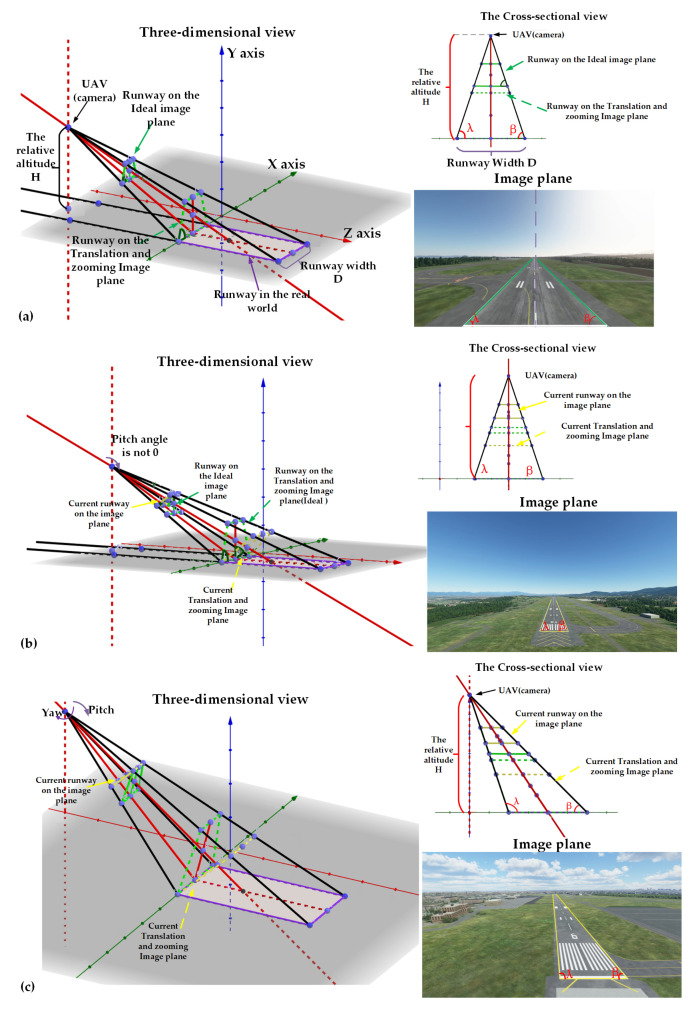
Different flight conditions and view of the altitude estimation model. (**a**) The Pitch, Yaw and Roll angles are $0^{\circ }$. (**b**) The Yaw and Roll angles are $0^{\circ }$, but Pitch is not $0^{\circ }$. (**c**) The Roll angle is $0^{\circ }$, but Pitch and Yaw angles are not $0^{\circ }$.

Figure 3 shows how the pose angles (Pitch, Yaw, Roll) impact the image plane and the altitude estimation model. The derivation of the ideal function starts from the flight state in which the pose angles are $0^{\circ }$. As shown in Figure 3a, following the principle of the pinhole camera and the perspective projection in [18], the altitude can be ideally represented as:

(2) $$Altitude=\frac{D\times \tan\lambda }{2},where\;the\;\lambda =\beta$$

When the pitch angle is not $0^{\circ }$, the cross-sectional view and image plane only have a slight change, but the perspective projection actually has a significant change which leads the bottom corners of the runway in the image plane λ and β to change into different values compared with the scene in Figure 3a. As the cross-sectional view shows in Figure 3b, after we projected the current image plane onto the ideal image plane by the pitch angle, the altitude can be ideally represented as:

(3) $$Altitude=\cos\left(Pitch\right)\times \frac{D\times \tan\lambda }{2},where\;the\;\lambda =\beta$$

When the pitch angle and relative yaw angle are not $0^{\circ }$, the image plane will significantly change, especially the values of λ and β. As shown in Figure 3c, the altitude should be represented as:

(4) $$Altitude=\cos\left(Pitch\right)\times \cos\left(Yaw\right)\times \frac{D}{\left(\frac{1}{\tan\lambda }+\frac{1}{\tan\beta }\right)},where\;the\;\lambda \neq \beta$$

Finally, we use a one-layer LSTM with 256 hidden states as the ideal altitude function encoder $f_{altitude}$. The number of hidden states is similar to the convolution channel size. If we set a larger value, the possibility of overfit will arise, and in contrast, if we set a smaller value, the model will meet an underfitting problem. Thus, it is difficult to decide the value unless we try it during our algorithm training. For our work, we have adapted empirical values from [48]. Although we have built an ideal physical model with different fixed-wing UAV poses, the function (4) does not take into account the effect of the roll angle. Moreover, the modeling of roll angle variation is a complex nonlinear problem that is difficult to represent as a linear function. Therefore, the proposed method uses the neural network to deal with this problem by using the IMU measurements to build an error function.

#### 3.1.2. Error Function

The ideal altitude function modeling process has shown that the pose angle is the most crucial factor for the image plane variation. Therefore, we assume that these pose angles cause the error between the ideal altitude model and the actual altitude model. Thus, the model of the proposed method can be represented as follows:

(5) F(Pitch,Yaw,Roll,λ,β)=f(Pitch,Yaw,Roll,λ,β)+ε(Pitch,Yaw,Roll)

We also use a one-layer LSTM with 256 hidden states as the error function encoder $f_{error}$. After the encoder processing, we add the ideal altitude function encoder results with the error function encoder results directly as the Formula (5) shows. Let $h_{f}$ and $e_{f}$ represent the ideal altitude function and error function for each image and IMU measurement, respectively. The function feature vector (in 8×512×1) can be represented as:

(6) $$a_{f}=f_{altitude}\left(h_{f}\right)+f_{error}\left(e_{f}\right)$$

### 3.2. Visual Feature Encoder

**Figure 4:**
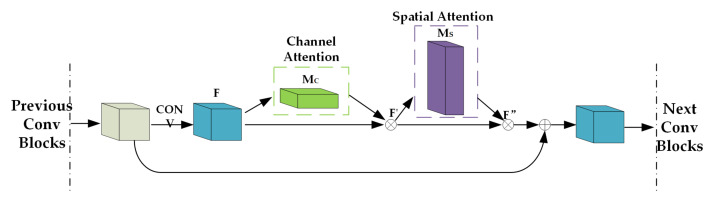
The channel attention and spatial attention integrated into ResNet-50 Block.

The visual encoder extracts latent dynamic information from a set of eight consecutive monocular images $v_{f}$. Ideally, we want the visual encoder to learn the common context or appearance features and focus on the important features (such as the runway on images). Inspired by previous works [48,49,50], we fed the images into the ResNet-50 [51] model which was pre-trained on the ImageNet [52] dataset, and added the convolutional block attention module [50] in ResNet-50. The convolutional block attention module (CBAM) was proposed to improve the representation ability of CNN networks. This attention module learns what and where to emphasize or suppress and refines intermediate features effectively. It has two sequential submodules: channel and spatial. Owing to these two submodules, the neural network can learn “what” and “where” to attend in the channel and spatial axes, respectively. The experiment results in [50] show that the channel-first order is slightly better than the spatial-first order. In our case, we want the visual feature encoder to focus on the runway in the sequence image data, thus we integrated this idea into our work. The convolutional block attention module integrated with ResNet-50 is shown in Figure 4.

Where $F\epsilon R^{C\times H\times W}$ represents the feature map. $F^{{}^{\prime }}$ represents the result of the feature map multiplied by the channel attention map, and $F^{{}^{\prime \prime }}$ represents the result of the spatial attention multiplied by $F^{{}^{\prime }}$. These processes can be represented as:

(7) $$F^{{}^{\prime }}=M_{C}\left(F\right)⊗F$$

(8) $$F^{{}^{\prime \prime }}=M_{S}\left(F^{{}^{\prime }}\right)⊗F^{{}^{\prime }}$$

In the end, we use the output feature vector (in 8×512×1) from the last FC layer as our visual feature:

(9) $$a_{v}=f_{vision}\left(v_{f}\right)$$

### 3.3. Visual-Inertial Feature Fusion

An elaborate feature fusion module is required to fuse different types of high-level features extracted from the visual and physical model encoders, especially considering the fact that the image and IMU measurement information are usually poorly calibrated and synchronized in practical applications. Thus, it is important to design a fusion function that combines different features that have different fundamental units. The previous work [41] directly concatenates the visual and inertial features into one feature space, which usually results in suboptimal performance. To help the network find the best suitable feature fusion, given visual-inertial feature inputs, we use a self-attention mechanism to reweight each feature inspired by the previous works [46,47,53]. Meanwhile, the feature fusion function is deterministic and differentiable, and can be jointly trained with other modules in the VIAE-Net.

**Figure 5:**
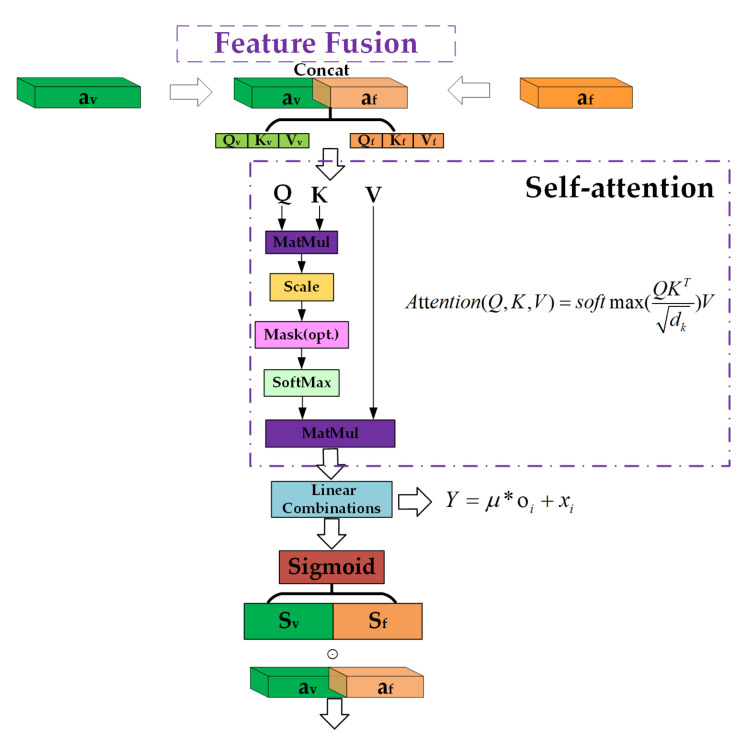
The proposed feature fusion structure.

As Figure 5 shows, the first step in the feature fusion module is directly concatenating the different types of feature vectors from encoders. Hence, the initial fusion is represented as:

(10) $$g_{dir}\left(a_{v},a_{f}\right)=\left[a_{v};a_{f}\right]$$

After the concatenating, we used scaled dot-product attention [46] to compute the similarity of $a_{v}$ and $a_{f}$. The $Q_{v}$, $K_{v}$ and $V_{v}$ are obtained by the input feature $a_{v}$ multiple with three different weight matrixes which can be learned during the algorithm training. We can treat the three vectors as a new representation of the input feature $a_{v}$. Meanwhile, the $Q_{f}$, $K_{f}$ and $V_{f}$ are vectors which represent the input feature $a_{f}$. This transformation aims to explore the feature association (similarity) between $a_{v}$ and $a_{f}$ (visual and physical model). The similarity can be modeled as:

(11) $$f\left(Q,K\right)=\frac{\left[Q_{v}^{T}K_{f},Q_{f}^{T}K_{v}\right]}{\sqrt{d}},where\;d\;is\;the\;dim\;of\;Q\;and\;K$$

Then, we used the softmax function to reweight the similarity value. The reweighted value can be modelled as:

(12) $$softmax\left(f\left(Q,K_{i}\right)\right)=\frac{exp(f\left(Q,K_{i}\right))}{\sum_{j}exp(f\left(Q,K_{j}\right)}$$

Finally, we used weighted summation with the reweighted value and the vector $V_{v},V_{f}$ to calculate the attention value. Thus, the attention value can be modelled as:

(13) $$Attention\left(Q,K,V\right)=\sum_{i}softmax\left(f\left(Q,K_{i}\right)\right)V_{i}$$

Here, the two types of features are mapped into the same feature space for learning the altitude estimation model. Before the output of self-attention is passed to temporal modeling and altitude regression, we design a residual structure like ResNet to emphasize the part of the feature that requires attention:

(14) $$y_{i}=\mu o_{i}+x_{i}$$

where $o_{i}$ is the output of self-attention and $x_{i}$ represents the input of self-attention, i.e., $a_{v}$ and $a_{f}$. To make sure that each of the features will be reweighted in the range [0, 1], we use the sigmoid function to process the $y_{i}$ and obtain the continuous masks $m_{v}$ and $m_{f}$ which are applied to visual features and inertial features, respectively, and deterministically parameterized by the neural networks:

(15) $$m_{v}=Sigmoid\left(y_{i}^{v}\left[a_{v};a_{f}\right]\right)$$

(16) $$m_{f}=Sigmoid\left(y_{i}^{f}\left[a_{v};a_{f}\right]\right)$$

Finally, the visual and inertial features are element-wise, multiplied with their corresponding masks at the new reweight vector. The fusion function is represented as:

(17) $$g_{fusion}\left(a_{v},a_{f}\right)=\left[a_{v}⨀m_{v};a_{f}⨀m_{f}\right]$$

### 3.4. Temporal Feature Extraction and Altitude Regression

Once the fused feature is obtained, the temporal feature extraction and altitude regression module serves to explore the temporal properties and regression relations for long-range altitude estimation. In recent years, researchers have found that recurrent neural networks (RNNs) have the advantage of a limited short-term memory. It is mainly because the RNNs contain internal cycles that feed the network activations from a previous state as inputs to the network to influence predictions at the current state. However, to face long sequence problems, gradient disappearance in backpropagation and gradient descent algorithms make the RNN training process difficult to converge. Inspired by the previous work in [44], a long short-term memory architecture was proposed to solve the gradient disappearance in a long sequence. Moreover, to fully consider the temporal characteristic of the sequence image and IMU data. A two-layer Bi-directional LSTM is connected behind the feature fusion. Unlike the common LSTM which only has a memory function for forward sequences, the Bi-LSTM can learn more temporal information from the sequence data by operating on the input sequence in both directions (forward and backward).

### 3.5. Model Training

The design, training, and evaluation of the altitude estimation model were embedded in the PyTorch framework. The training was done with the dataset obtained by a fixed-wing UAV in the landing phase. The dataset includes 2400 images and the corresponding IMU measurements. We have split the whole dataset into a training set (80% of the dataset) and a validation set (20% of the dataset).The dataset was randomized by shuffling and was fed into batches of size 64. The model was trained for up to 300 epochs on the training datasets. For the model design and fitting, we adopted an Adam optimizer with an initial learning rate of 0.0005, which decayed at a rate of 0.9 after every 20 epochs.The loss function was L1-smooth which combines the advantages of L1-loss (steady gradients for large values of *x*) and L2-loss (fewer oscillations during updates when *x* is small):

(18) $$L_{1smooth}=\left\{\begin{array}{c} \left|x\right|\\ \frac{1}{\left|\alpha \right|}x^{2} \end{array}\right.$$

To accelerate the training, we used four NVIDIA GTX TITAN X GPUs and a multi-GPU training mechanism in PyTorch. All of the hyper-parameters inside the networks were identical for a fair comparison.

## 4. Results Comparisons and Analysis

In this section, we first introduce the real datasets used in the experiments and the details of experimental implementation, then present the altitude estimation results on the different sequence data during the auto-landing phase from the real fixed-wing UAV. Moreover, we provide qualitative and quantitative comparisons between our proposed method and state-of-the-art methods.

### 4.1. Experimental Data and Metrics

**Figure 6:**
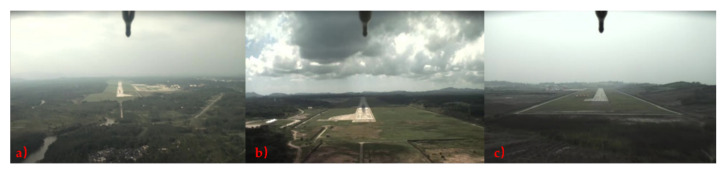
The images captured at different flight altitudes during the UAV landing phase. (**a**) The image is captured in the altitude range of 160~110 m. (**b**) The image is captured in the altitude range of 105~75 m. (**c**) The image is captured in the altitude range of 70~40 m.

**Datasets:** To better evaluate the proposed method, we establish a visual-inertial database in the real environment. This database provides abundant visual and inertial data captured by a front-view camera and inertial measurement units in real UAV landing scenarios. In other words, the captured data have motion blur, jitter of the UAV, and inertial information drift, which are great challenges for algorithm robustness. A front-view camera captures the visual images (in size 1280×720) in video format. Some samples of the image data are shown in Figure 6. The IMU measurements are captured by the INS on the fixed-wing UAV at 17 fps. All of the ground-truth altitudes required for algorithm training, validation and testing are obtained from the real-time kinematic global positioning system (RTK-GPS) on board the UAV [54,55]. We did a coarse data synchronization and kept the images’ frame rate the same as the IMU measurements. The training data includes 2400 images and IMU measurements, and the test data includes 600 images and IMU measurements, the training data and test data are independent of each other.

**Figure 7:**
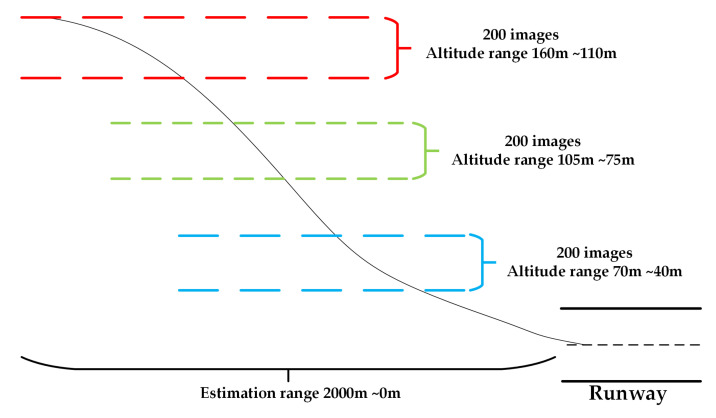
The test images are selected from different scenes at different flight altitudes.

To evaluate the methods’ performance at different flight altitudes, we split the test data based on different range flight altitudes as the Figure 7 shows below:

**Metrics:** In order to compare the performance of the different methods, the work uses well-known and widely-used evaluation metrics: The mean-absolute error (MAE), the Root-Mean-Squared Error (RMSE), and the Pearson correlation score. Let *D* be a set, *h* be the trained model, *x* be the input data or feature and *y* be the true altitude. The MAE is calculated using the following formula:

(19) $$MAE\left(h,D\right)=\frac{1}{\left|D\right|}\sum_{(x,y)\epsilon D}\left|h\left(x\right)-y\right|$$

Regarding the RMSE, as for the MAE, the smaller its value is, the more accurate the estimation is. The RMSE is calculated using the following formula:

(20) $$RMSE\left(h,D\right)=\sqrt{\frac{1}{\left|D\right|}\sum_{(x,y)\epsilon D}{\left(h\left(x\right)-y\right)}^{2}}$$

The coefficient of determination is a statistical measure used to indicate how close the regression predictions approximate the real data points. The closer the score is to 1, the better is the estimation. It was calculated using the following equation:

(21) $$R^{2}=1-FUV=1-\frac{RSS}{TSS}=1-\frac{\sum_{i}{\left(y_{i}-h\left(x\right)\right)}^{2}}{\sum_{i}{\left(y_{i}-{\hat{y}}_{i}\right)}^{2}}$$

where the FUV is called the fraction of unexplained variance, the RSS means the residual sum of squares, and TSS means the total sum of squares.

### 4.2. Experimental Results and Analysis

The proposed method was compared to different types of existing works for altitude estimation, which include the traditional visual method in [56], the traditional visual-inertial method in [36], and the deep learning method in [41,44]. As we mentioned above, the pure monocular methods, such as [44,56], cannot obtain the altitude estimation directly. Thus, we also introduced the true width of the runway into the algorithm implementation. Moreover, the visual-inertial methods [36,41] were not designed for altitude estimation. Thus, for obtaining the regression of altitude and a fair comparison, we modified part of the implementation and added the true width of the runway as well. Before the comparison, we used object detection [57] and line feature detection algorithms [58] to detect the runway on the image plane, which is required by all of the methods to obtain the altitude estimation value in the real world, and then used the detected results to compute the bottom angles, which are required for the ideal altitude estimation function in our method as additional visual information. The relative heading angle yaw was calculated based on the UAV heading angle and the records of the runway orientation angle. This is the relative pose angle required by all of the comparison methods.

The results in Table 2, Table 3 and Table 4 show that the performance of the traditional visual method gets better when the image is captured close to the runway, which means that the image quality is better and the motion of the UAV is getting smooth. Moreover, the modeling process without the intrinsic camera calibration will impact on the prediction’s stability and accuracy.

Visual-inertial methods have been proposed for robot navigation tasks in recent years to solve motion blur and drift IMU measurements. These applications on small UAVs or drones almost solved the automatic landing problem with high precision. However, the good performance is based on fine calibration and synchronization for the visual-inertial system. Thus, as the results in Table 2, Table 3 and Table 4 show, the traditional visual-inertial methods with coarse calibration and synchronization can improve the estimation results when the UAV is far from the runway or in aggressive UAV motion scenarios. However, even when the UAV is close to the runway, errors from rough calibration and synchronization cannot be eliminated. Therefore, the average error of altitude estimation is even higher than the traditional visual methods when the UAV is closer to the landing area.

As the results in Table 2, Table 3 and Table 4 show, the main advantage of learning-based approaches is their potential capability to perceive the implicit data association from the data sequences and use the data association to build a better altitude model to obtain accurate altitude estimation results. Benefits arise from these advantages, when facing inaccurate sensor calibration and synchronization, motion blur, and the difficulty of kinematic modeling, where deep learning methods show a more robust and accurate performance than traditional methods. Especially, our method is significantly superior to the other methods.

**Figure 8:**
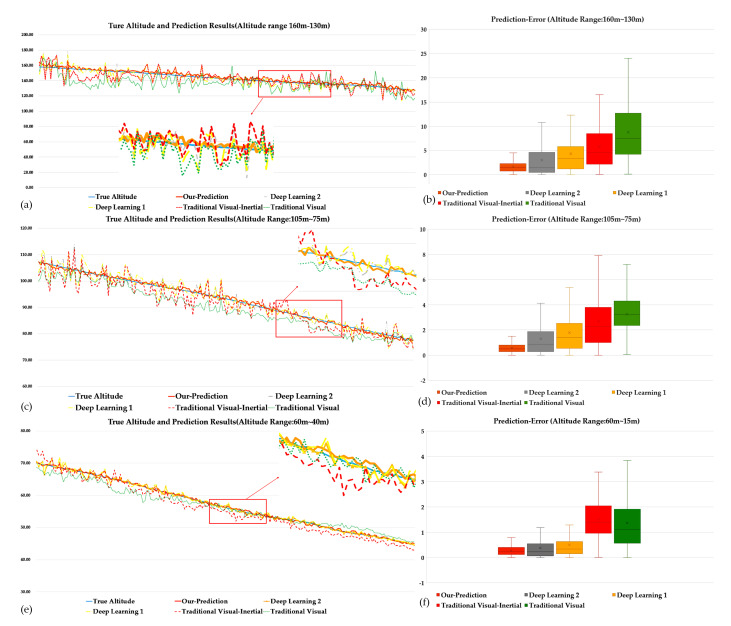
The comparison of all methods’ prediction results and the prediction−error. (**a**) shows the prediction results of all the methods in altitude range from 160 m to 130 m. (**b**) shows the prediction error of all the methods in altitude range from 160 m to 130 m. (**c**) shows the prediction results of all the methods in altitude range from 105 m to 75 m. (**d**) shows the prediction error of all the methods in altitude range from 105 m to 75 m. (**e**) shows the prediction results of all the methods in altitude range from 60 m to 40 m. (**f**) shows the prediction error of all the methods in altitude range from 60 m to 40 m.

To visually assess the effect of our method and the others, in Figure 8, we compare these methods by showing the actual flight altitude values and these methods’ predicted altitude values in different ranges of flight altitude. The results are shown in Figure 8a–f.

Figure 8a,b shows that the traditional visual method cannot obtain precise and robust altitude estimation results since the UAV is far from the landing area, and the quality of the captured images can be easily impacted by the jitter and motion of the UAV. At the same time, when the method compensates the inertial information with the visual information, the altitude estimation results get better than the traditional visual method since the visual information was blurred or missing. The shown prediction results of deep learning methods are significantly robust and accurate, and are based on the perception of the parameters from the visual and inertial data sequences to build the implicit altitude estimation model. Especially, our method that combines the physics-based model with the learning-based perception of the parameters demonstrates the best performance compared to the other methods, in the average prediction error and peak error.

Figure 8c,d shows that when the UAV is getting closer to the landing area, the capture of visual and inertial information becomes more stable, and the quality of the visual information becomes better than previously. Therefore, all of the methods’ performances are significantly improved. However, the robustness of the traditional visual-inertial method becomes worse than the traditional visual method, which is caused by the obtained inaccurate data association from the coarse visual and inertial sensors calibration and synchronization. Compared to other methods, our method still shows significant advantages in performance.

Figure 8e,f shows that when the data capture becomes accurate and stable as the UAV is near the landing area, the robustness and precision of these altitude estimation methods achieve their best performance. As the prediction error shows, the coarse visual and inertial sensors calibration and synchronization still impacts the traditional visual-inertial method performance. In contrast, the average prediction error of our method is getting to less than 0.5 m. At the same time, the peak error of our method is significantly better than the other compared methods.

**Figure 9:**
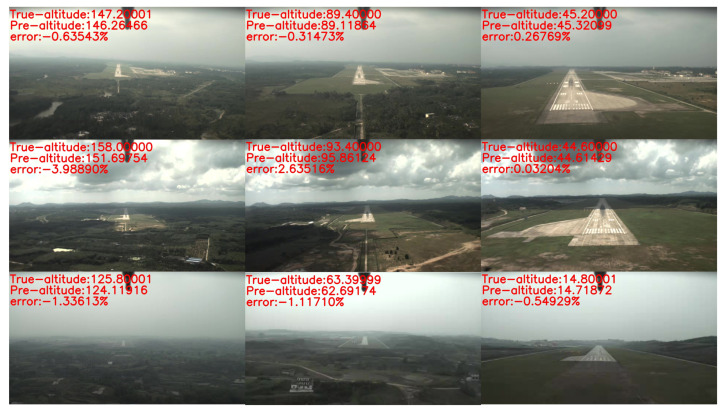
The altitude estimation results in real landing scenes.

Figure 9 shows our altitude estimation results in real landing scenes. Each of the rows shows the image captured by the monocular camera in different scenes, the true altitude from the GPS when the image is captured, the predicted altitude from our method, and the prediction error of our method. These results demonstrated that our method could provide precise and robust altitude estimation at a long-range, even when the quality of the data sequences is poor.

We also tested the operating time of our algorithm on the computer with the NVIDIA GTX TITAN X GPU. The time cost of a single frame is near 0.1–0.11 s (excluding the time cost of the runway detection and line feature detection). In other words, the algorithm can reach a 10 frames per second (fps) operating speed. Moreover, even when we added the runway detection and line feature detection for testing, the operating speed can reach near 4 fps (time cost of a single frame is near 0.24 s). We also tested our algorithm on the embedded hardware platform (NVIDIA JETSON XAVIER NX); the operating speed can reach near 2.5 fps (time cost of a single frame is near 0.4 s). For the embedding hardware platform, we still need to do a lot of work (such as model pruning and quantization for deep learning model compression) to improve our algorithm to reach real-time application requirement (10 fps).

## 5. Conclusions

Solving the task for altitude estimation of fixed-wing UAVs, this study presented an end-to-end neural network architecture with a novel physical-based model to improve its applicability and data efficiency. Meanwhile, a novel feature fusion module is designed to fuse the different features that represent the visual information and the physical-based model and help the neural network to explore high-level data associations to obtain a robust and accurate altitude estimation model. To evaluate the performance of our system, we conducted a landing experiment with fixed-wing UAVs in the real scenario, including 600 different position samples (relative distance and altitude between the UAV and runway) from the UAV landing phases. The experimental results show that our method achieves a minimal peak error (near 5%) and mean error (less than 3%) compared to the other methods. In addition, extensive experiments show that our model achieves better performance than state-of-all-art models with different UAV flight positions. As one can see, the RMSE of our model surpasses that of the state-of-the-art model by up to five times. In the meantime, the coefficient of determination further increases by a large margin (up to 10%).

## 6. Future Work

It is known that the conventional camera cannot work in bad visibility conditions, such as limited visibility, dusk, sunset, night time etc. However, we can use an infrared camera instead of it. Therefore, The proposed method can be adapted and potentially applied to infrared images, with minor modifications of the visual encoder module. On the other hand, if the optical sensor cannot observe landing area objects, we may use the measurement or information of the other sensors (such as barometric altimeter, and GPS) to fuse with the inertial information to achieve an accurate altitude estimation. In other words, with slight modifications the feature can be applied for multimodal data fusion which will drive the deep neural network to learn a robust and accurate altitude estimation model for fixed-wing UAVs. Moreover, as the proposed method only focused on the altitude, we only considered the relevant physical principles while designing the algorithm. However, accurate UAV landing or navigation tasks require a more a comprehensive state estimation. Therefore, future work can be carried out modifying parts of the physical-based model design. We will first design a more comprehensive kinematic model to represent the location and pose of the landing for fixed-wing UAVs, and then integrate the new kinematic model into the deep neural network to obtain a robust and accurate model for the 6-Dof (degrees of freedom) state estimation.

## Figures and Tables

**Table 1:** The main properties of Physical-based methods and Learning-based methods.

Method	Physical-Based	Learning-Based
	Good generality	Highly accurate in trained regime
**Advantage**	Physics are universal	Highly robust in trained regime
	Data efficient	Require little priors
	Require strong priors assumption	Require large amount of data
**Disadvantage**	Require good modeling	Risk of overfitting
	Hardly achieve better accuracy	Generality only in trained regime

**Table 2:** The MAE (in meters), RMSE (in meters) and coefficient of determination ($R^{2}$) results on the test data with the 110~160 m range of flight altitude are reported.

Method	MAE (m)	RMSE	$R^{2}$
Traditional Visual [56]	7.6760	11.1068	−1.1551
Traditional Visual-Inertial [36]	6.0696	9.4284	−0.5157
Deep Learning 1 [44]	3.9028	5.1624	0.5954
Deep Learning 2 [41]	3.1246	4.1994	0.7327
**Our Method**	**1.0567**	**1.1866**	**0.9679**

**Table 3:** The MAE (in meters), RMSE (in meters) and coefficient of determination ($R^{2}$) results on the test data with the 70~105 m range of flight altitude are reported.

Method	MAE (m)	RMSE	$R^{2}$
Traditional Visual [56]	5.9274	6.6192	0.5763
Traditional Visual-Inertial [36]	3.2191	3.8659	0.7414
Deep Learning 1 [44]	1.7654	2.5537	0.8985
Deep Learning 2 [41]	1.3243	2.1242	0.9288
**Our Method**	**0.3402**	**0.4688**	**0.9961**

**Table 4:** The MAE (in meters), RMSE (in meters) and coefficient of determination ($R^{2}$) results on the test data with the 15~60 m range of flight altitude are reported.

Method	MAE (m)	RMSE	$R^{2}$
Traditional Visual [56]	1.7055	2.0385	0.9189
Traditional Visual-Inertial [36]	4.4189	4.0917	0.6754
Deep Learning 1 [44]	0.4303	0.6304	0.9921
Deep Learning 2 [41]	0.3443	0.5287	0.9961
**Our Method**	**0.1368**	**0.1715**	**0.9993**
